# Characteristics of Polybenzoxazine Aerogels as Thermal Insulation and Flame-Retardant Materials

**DOI:** 10.3390/gels11020121

**Published:** 2025-02-06

**Authors:** Shakila Parveen Asrafali, Thirukumaran Periyasamy, Jaewoong Lee

**Affiliations:** Department of Fiber System Engineering, Yeungnam University, Gyeongsan 38541, Republic of Korea; shakilaparveen@yu.ac.kr (S.P.A.); thirukumaran@ynu.ac.kr (T.P.)

**Keywords:** PBZ aerogels, synthesis procedure, thermal insulation, lightweight, enhanced mechanical strength

## Abstract

Polybenzoxazine-based aerogels are a unique class of materials that combine the desirable properties of aerogels—such as low density, high porosity, and excellent thermal insulation—with the outstanding characteristics of polybenzoxazines—such as high thermal stability, low water absorption, and superior mechanical strength. Polybenzoxazines are a type of thermosetting polymer derived from benzoxazine monomers. Several features of polybenzoxazines can be retained within the aerogels synthesized through them. The excellent thermal resistance of polybenzoxazines, which can withstand temperatures above 200–300 °C, makes their aerogel able to withstand extreme thermal environments. The inherent structure of polybenzoxazines, rich in aromatic rings and nitrogen and oxygen atoms, imparts flame-retardant property. Their highly crosslinked structure provides excellent resistance to solvents, acids, and bases. Above all, through their molecular design flexibility, their physical, mechanical, and thermal properties can be tubed to suit specific applications. In this review, the synthesis of polybenzoxazine aerogels, including various steps such as monomer synthesis, gel formation, solvent exchange and drying, and finally curing are discussed in detail. The application of these aerogels in thermal insulation and flame-retardant materials is given importance. The challenges and future prospects of further enhancing their properties and expanding their utility are also summarized.

## 1. Introduction

Flame retardants (FRs) are chemicals designed to reduce the flammability of materials, either by making them less likely to catch fire or by slowing the spread of flames. They are widely used across various industries to enhance fire safety [1,2,3]. Applications include furniture and textiles (applied to foam, fabric, and coatings to meet flammability standards), electronics (incorporated into circuit boards, casings, and cables), building materials (such as insulation, paints, and coatings), and transportation components (used in automotive and aviation materials) [4,5,6,7]. FRs are categorized based on their chemical structure and applications, including the following: Brominated Flame Retardants (BFRs): Commonly used in electronics and textiles, BFRs are highly effective but linked to potential health risks; Phosphorus-Based Flame Retardants (PFRs): Found in plastics, foams, and textiles, PFRs include organophosphates and inorganic phosphorus compounds; Chlorinated Flame Retardants (ClFRs): Used in certain fabrics and foams, ClFRs share similar health concerns with BFRs; Inorganic Flame Retardants (IoFRs): These include aluminum hydroxide, magnesium hydroxide, and antimony trioxide. While safer, they may be less effective in some applications; Nitrogen-Based Flame Retardants (NFRs): Primarily used in specialized applications like textiles, NFRs have a low environmental impact [8,9,10,11]. Certain flame retardants, particularly brominated flame retardants (BFRs) and specific organophosphates, have been associated with health concerns such as endocrine disruption, developmental delays, and an increased risk of certain cancers. Additionally, many of these compounds are resistant to degradation, leading to their accumulation in ecosystems and entry into the food chain. To address these issues, efforts to develop safer alternatives focus on the following aspects: (i) Non-Toxic Additives: Utilizing inorganic compounds or bio-based materials to enhance fire resistance without harmful effects; (ii) Innovative Coatings: Creating advanced solutions like nano-coatings and intumescent coatings that improve fire resistance; and (iii) Fire-Resistant Materials: Developing fibers and plastics that are inherently resistant to fire. These approaches aim to balance fire safety with environmental sustainability and human health [12,13,14,15].

**Figure 1:**
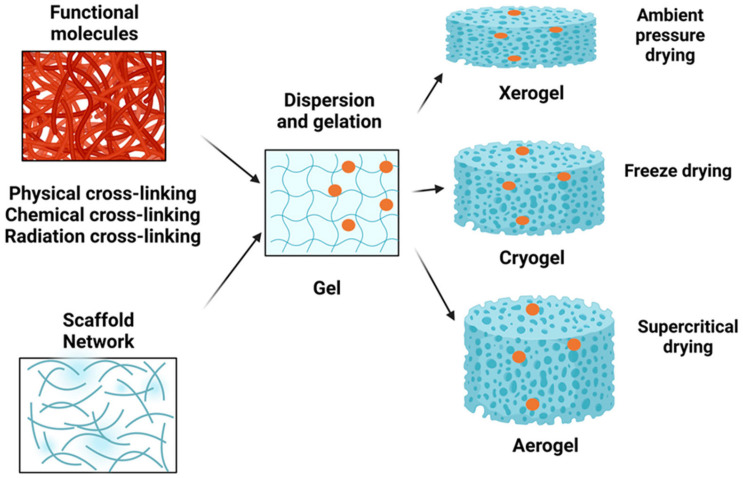
Different procedures for obtaining xerogels, cryogels, and aerogels [71].

Traditional flame retardants often rely on halogens or other chemicals that pose health and environmental risks. They may also be heavy or degrade material properties, which could be effectively overcome by aerogels. Aerogels are ultra-lightweight, highly porous materials with outstanding thermal, mechanical, and chemical properties, making them increasingly significant in flame retardancy applications (Figure 1) (Table 1). Their growing importance stems from their ability to overcome the limitations of conventional flame-retardant materials while providing unique advantages [16,17,18,19,20,21]. As some of the best thermal insulators, aerogels effectively reduce heat transfer during combustion. Their exceptional insulating properties prevent heat penetration, safeguarding underlying materials from high temperatures. The porous structure of aerogels offers a large surface area for interaction with flames, facilitating the formation of a protective char layer. Additionally, their porosity enhances the barrier effect by minimizing the diffusion of heat, oxygen, and flammable gases. Aerogels are extremely lightweight, making them ideal for applications where weight is critical, such as in aerospace, automotive industries, and wearable textiles [22,23,24,25,26]. They provide flame-retardant capabilities without significantly adding to material weight. Despite their light and porous structure, aerogels maintain good mechanical strength, enabling them to function effectively as both insulation and structural components. Their durability ensures long-lasting fire protection across various applications. Many aerogels are derived from silica or bio-based materials, making them non-toxic and environmentally friendly. Unlike traditional flame retardants, they do not release harmful gases during decomposition, aligning with the increasing demand for sustainable and safe fire-resistant solutions. Advances in aerogel technology, such as hybrid and polymer-reinforced aerogels, have further enhanced their flame-retardant potential by improving mechanical strength, thermal stability, and compatibility with various substrates. By combining high-performance fire protection with environmental safety and versatility, aerogels represent a promising solution for modern flame-retardant applications [27,28,29,30,31,32].

Polybenzoxazines (PBZs) are a class of thermosetting, non-halogen polymers that have attracted significant attention as flame retardants due to their unique properties. Their molecular structure and curing behavior make them highly effective for enhancing fire resistance in a wide range of materials [33,34,35,36,37]. The key characteristics of PBZs as flame retardants include excellent thermal stability, low flammability, high char yield, halogen-free composition, good processability, and enhanced mechanical and chemical robustness. The benzoxazine rings in PBZs decompose under heat to form crosslinked networks and carbonaceous char, providing flame-retardant properties without the need for additional additives. Moreover, PBZs produce substantial residue during thermal decomposition, which is a critical factor in suppressing fire propagation and enhancing flame retardancy [38,39,40,41,42,43,44,45,46,47,48,49,50]. PBZs have low viscosity before curing, allowing for easy processing and uniform application in various materials, including composites, textiles, and coatings. Unlike many traditional flame retardants, PBZs do not contain halogens, which reduces the release of toxic or corrosive gases during combustion. This feature makes them safer for human health and more environmentally friendly. Their aromatic backbone and strong covalent bonds, resisting degradation even at elevated temperatures, makes them ideal for high-performance applications. PBZs retain their mechanical properties under extreme conditions, ensuring durability. They are also resistant to moisture and chemicals, contributing to long-term performance [51,52,53,54]. Polybenzoxazines are highly promising due to their combination of flame-retardant efficiency, environmental friendliness, and versatility in application. Different forms of PBZs are used in flame-retardant applications: For example, PBZ composites with improved fire resistance, are used in aerospace, automotive, and construction materials; PBZ coatings are applied to textiles, electronics, and building materials to enhance flame-retardant properties; and PBZ foams are incorporated into insulation and cushioning materials for fire safety [55,56,57,58,59,60,61,62,63,64,65,66,67,68,69,70].

**Figure 2:**
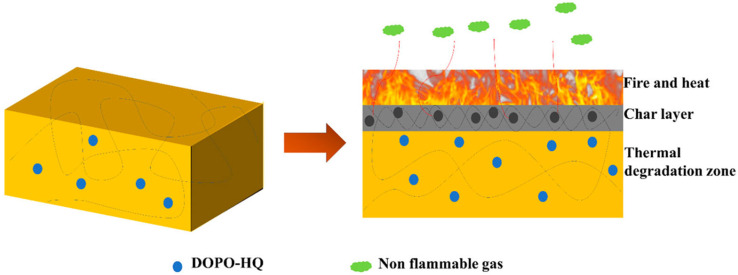
Possible flame-retardant mechanism of PBZ aerogels [82].

Polybenzoxazine aerogels emerged as innovative solutions for thermal protection systems, with the groundwork in benzoxazine chemistry for aerogel synthesis laid in the early 2000s. Initial efforts focused on creating lightweight, durable materials for insulation and structural applications (Figure 2). By the mid-2010s, advancements in pyrolysis processes transformed polybenzoxazine aerogels into carbon aerogels, characterized by their high porosity and electrical conductivity, making them ideal for use in electrodes and sensors [71,72,73,74,75,76,77,78,79,80,81,82]. To enhance flame retardance and thermal insulation, silicon and boron-doped polybenzoxazine aerogels and related derivatives were developed [8,27,81], showcasing exceptional performance in demanding environments, including aerospace and industrial sectors. More recently, research has pivoted toward hybrid systems, combining polybenzoxazine aerogels with materials such as silica and fabric reinforcements to further optimize their mechanical and thermal properties [12,82]. This review offers comprehensive insights into the characteristics of polybenzoxazine aerogels and their development as flame-retardant materials. Despite significant advancements in the development of PBZ materials, critical knowledge gaps remain, which must be addressed in order to enhance their application as flame-retardant materials. This review highlights key areas of focus, including the optimization of synthesis routes, improving processability, and functionalizing PBZ for advanced applications. It also examines the thermal and mechanical behavior of PBZ under extreme conditions and provides an overview of recent research on PBZ aerogels for thermal insulation. Additionally, it discusses the challenges that persist in optimizing PBZ aerogels, aiming to unlock their full potential for diverse advanced applications.

## 2. Polybenzoxazine Aerogels Derived from Bisphenol-A

**Figure 3:**
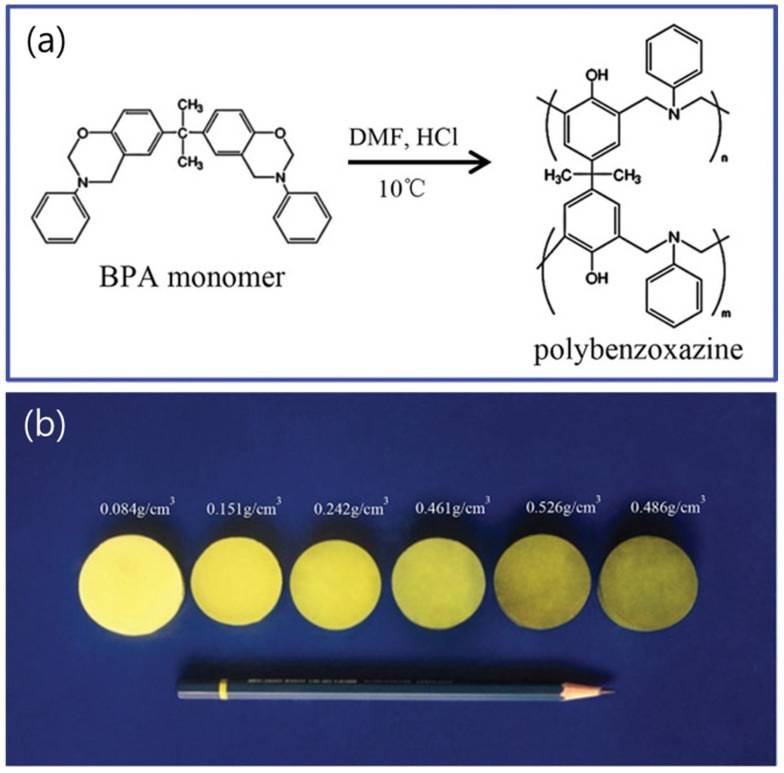
(**a**) Scheme showing the synthesis of PBO aerogels and (**b**) specimens of PBO aerogels [38].

**Figure 4:**
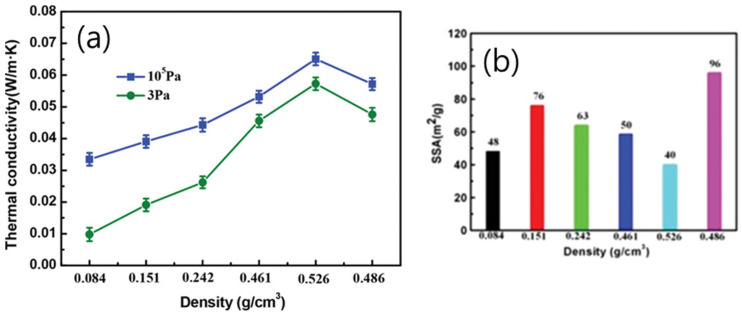
(**a**) Thermal conductivities and (**b**) specific surface areas of PBO aerogels [38].

Xiao et al. [38] developed polybenzoxazine (PBO) aerogels with low densities and thermal conductivities using bisphenol A (BPA) benzoxazine monomers at varying concentrations (Figure 3). The synthesis involved ring-opening polymerization catalyzed by HCl at 10 °C. The resulting PBO aerogels exhibited crosslinked, three-dimensional network structures with densities ranging from 0.084 to 0.526 g/cm^3^. Their thermal conductivities ranged from 0.0335 to 0.0652 W/m·K under ambient pressure and from 0.0098 to 0.0571 W/m·K at 3 Pa (Figure 4). Thermal conductivity decreased with increasing gas molecular weight across various atmospheres, including N_2_, Ar, and CO_2_. While conductivity showed minimal variation between 3 and 1000 Pa, it increased significantly beyond 1000 Pa as air pressure rose. Compared to conventional organic foam insulation materials like phenolic foam, polyurethane, and polystyrene, PBO aerogels demonstrated notably lower densities (as low as 0.084 g/cm^3^) and superior thermal insulating properties, with the lowest thermal conductivity measured at 0.0335 W/m·K.

**Scheme 1:**
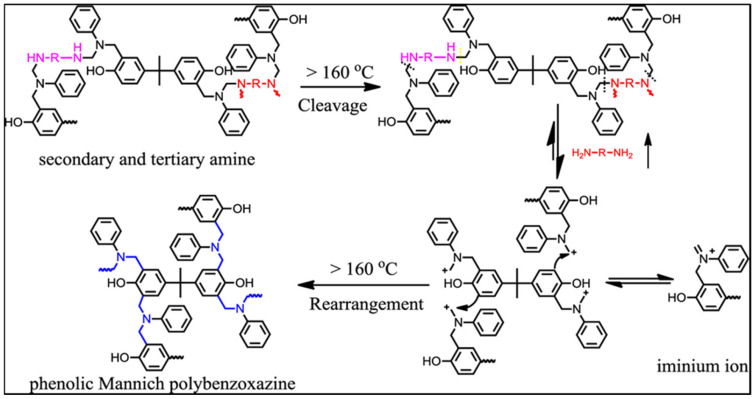
Proposed cleavage and rearrangement path of BA-a/hexamethylenediamine copolymer [50].

Zhang et al. [50] developed lightweight, robust, and thermally stable polybenzoxazine foams using a straightforward sol–gel method (Scheme 1). The process employed a commercially available benzoxazine monomer (BA-a) with hexamethylenediamine as the curing agent and methanol/chloroform as the solvent, eliminating the need for supercritical or freeze drying. By varying the initial concentrations of the organic solutions, foams with densities of 0.2815 g/cm^3^ (F-20), 0.4798 g/cm^3^ (F-30), and 0.6731 g/cm^3^ (F-40) were obtained.

**Figure 5:**
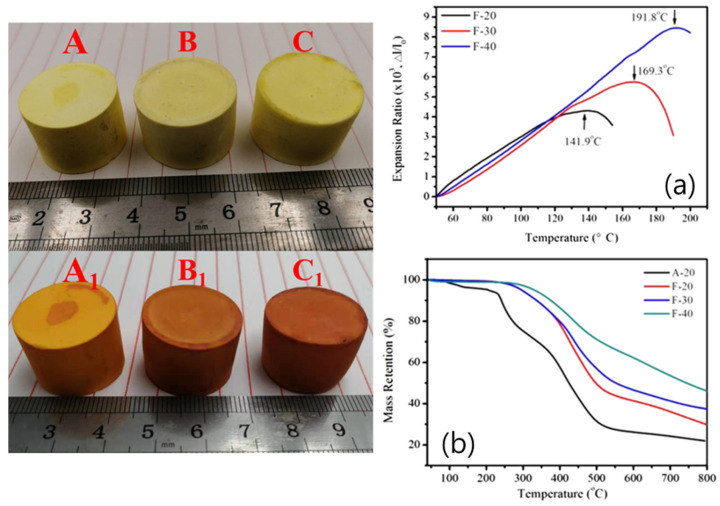
[**Left**] Photographs of freestanding aerogels: A-20 (A), A-30 (B), and A-40 (C) obtained by the removal of organic solvents at 30 °C, polybenzoxazine foams: F-20 (A1), F-30 (B1), and F-40 (C1) after curing at 200 °C; [**Right**] (**a**) TMA and (**b**) TGA thermograms of polybenzoxazine foams [50].

Scanning electron microscopy (SEM) images revealed that both A-20 (PBZ aerogel with 20 wt.% of benzoxazine monomer) and F-20 (PBZ foam with 20 wt.% of benzoxazine monomer) were open-cell foams featuring sparse rod-like walls and pore sizes ranging from 10 to 50 μm. The open-cell structure played a crucial role in facilitating the rapid evaporation and removal of organic solvents. Unlike the randomly overlapped linear structures of A-20, the pore walls of F-20 were fused into branched networks, enhancing mechanical strength. The porous structure of the foams contributed to their outstanding properties, including a low dielectric constant (1.47 at 1 MHz for F-20), low dielectric loss (0.003 at 1 MHz for F-20), low thermal conductivity (0.0604 W/m·K for F-20), and a low coefficient of linear expansion (55.1 ppm/°C for F-20). Foam F-40 exhibited a glass transition temperature (T_g_) of 191.8 °C, a char yield of 46.1%, and a compressive strength of 71.74 MPa (Figure 5). This study demonstrated that by adjusting the initial solution concentrations, the pore structure and properties of polybenzoxazine foams could be tailored efficiently, providing a versatile approach for designing foams with specific characteristics.

**Figure 6:**
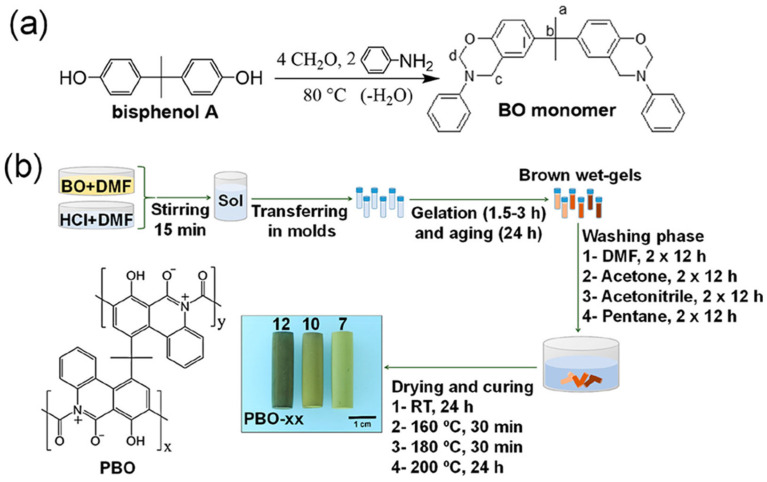
(**a**) Chemical synthesis of the benzoxazine (BO) monomer. (**b**) Synthetic protocol of the ambient-dried polybenzoxazine (PBO) aerogels [65].

Malakooti et al. [65] developed a series of high-performance polybenzoxazine (PBO) aerogels using a straightforward, scalable, ambient-drying process (Figure 6). These aerogels exhibited robust thermo-mechanical properties at elevated temperatures, inherent flame retardancy, and superhydrophobicity across their entire bulk density range (0.24–0.46 g/cm^3^). Notably, they demonstrated superior mechanical strength, such as a compressive strength of 1 MPa at 0.24 g/cm^3^ at room temperature, outperforming other high-performance aerogels of similar density, including polyimide, polyamide (Kevlar-like) aerogels, and polymer crosslinked X-silica and X-vanadia aerogels, all at significantly lower costs. The mechanical resilience of these aerogels under high-temperature conditions was evaluated through quasi-static compression experiments at varying densities, confirming their exceptional thermal stability. The infrared imaging of the lowest-density aerogels on a heat stage revealed that the temperature gradient across the sample grew for the first 10 s and then stabilized after 1 min. Additionally, the aerogels demonstrated thermal conductivities ranging from 0.097 W/m·K (for PBO-7) at the lowest density to 0.13 W/m·K (for PBO-12) at the highest density, emphasizing their suitability for thermal insulation applications.

**Figure 7:**
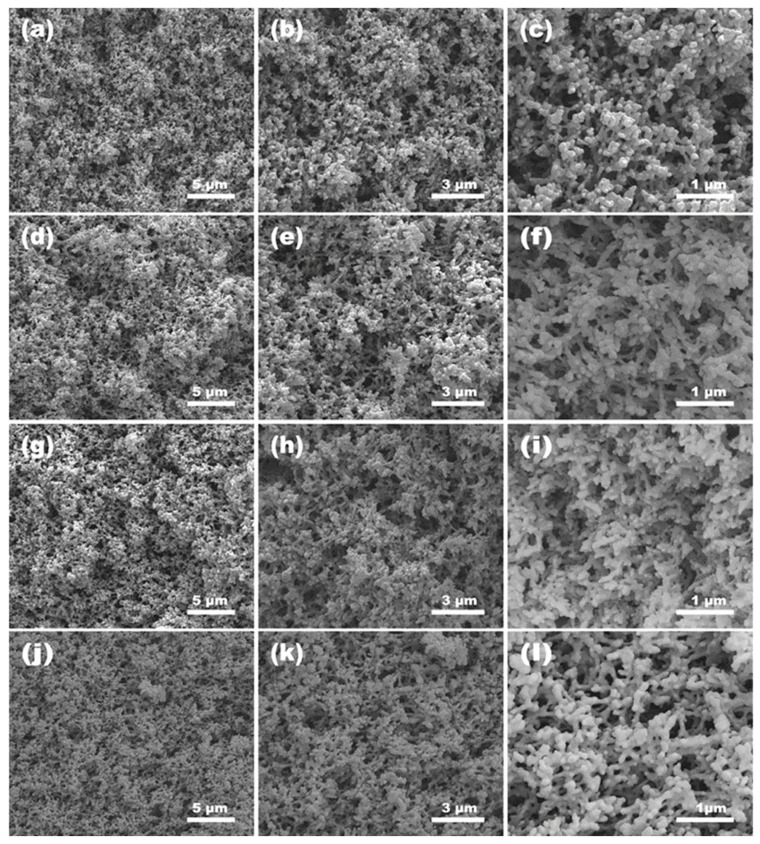
FESEM images of (**a**–**c**) PBO-FT, (**d**–**f**) PBO-AH, (**g**–**i**) PBO-AE, and (**j**–**l**) PBO-AT under different magnifications [1].

Xiong et al. [1] synthesized nanoporous polybenzoxazine (PBO) aerogels with exceptional thermal insulation and self-extinguishing properties using a cost-effective ambient pressure drying (APD) method. This approach eliminates the need for conventional high-pressure supercritical drying. The PBO aerogels were prepared using different exchange solvents, including tert-butyl alcohol, ethanol, and n-hexane. All PBO aerogels exhibited a 3D nanonetwork structure having a good skeletal strength due to gelling and aging stages. It was found that different solvents and drying methods did not have any influence on the dimensions of the PBO aerogels. The maximum pore size was around 150 nm, with more pores between 60 and 70 nm (Figure 7). Among these, PBO-AH, created using n-hexane as the exchange solvent, demonstrated a more uniform particle and pore size structure. The nonpolar nature of n-hexane effectively reduced surface tension between the gel and solvent during drying, resulting in a well-preserved, uniform network structure.

**Figure 8:**
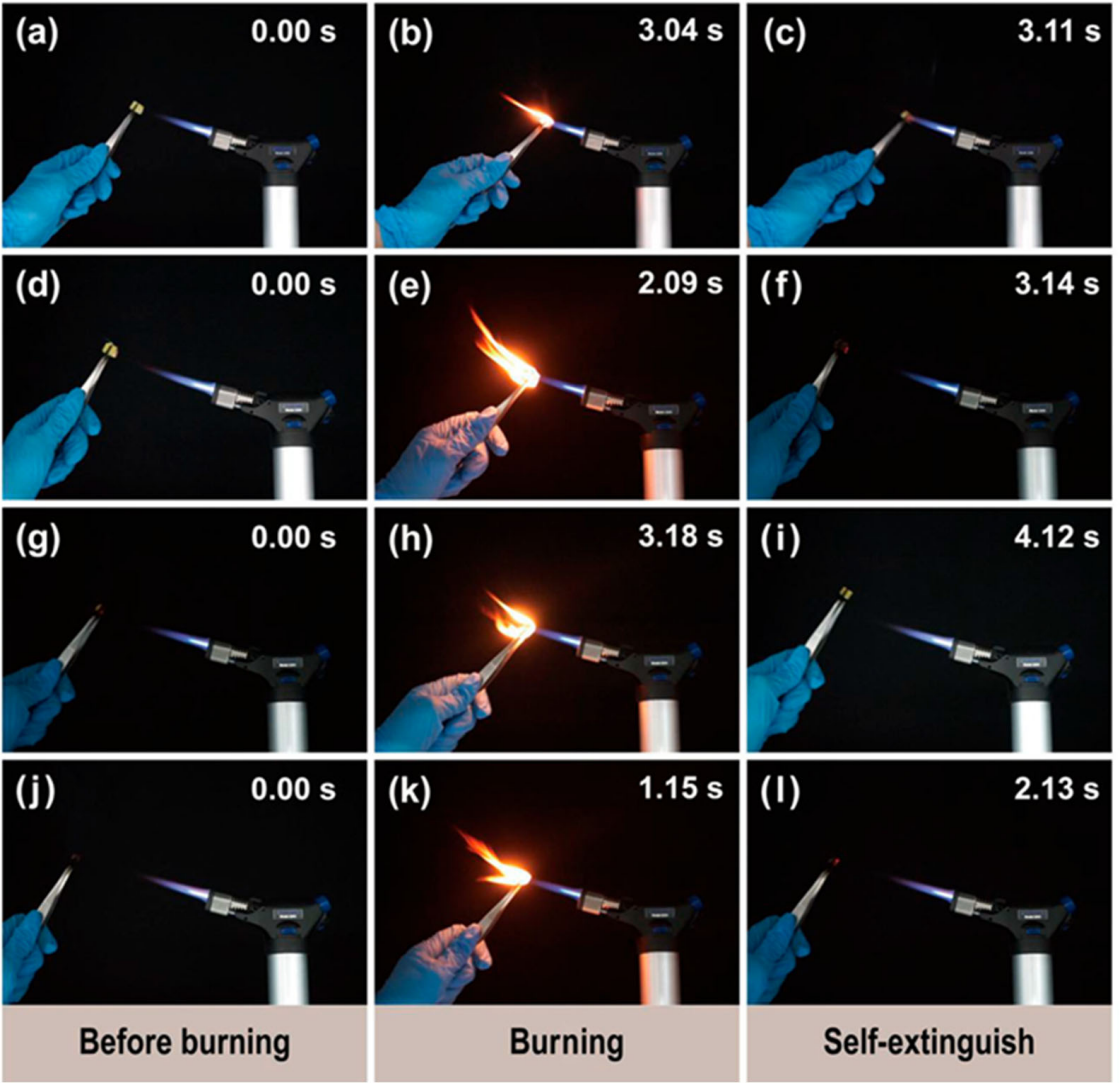
Self-extinguishing tests of PBO aerogels (**a**–**c**) PBO-FT, (**d**–**f**) PBO-AH, (**g**–**i**) PBO-AE, and (**j**–**l**) PBO-AT [1].

**Figure 9:**
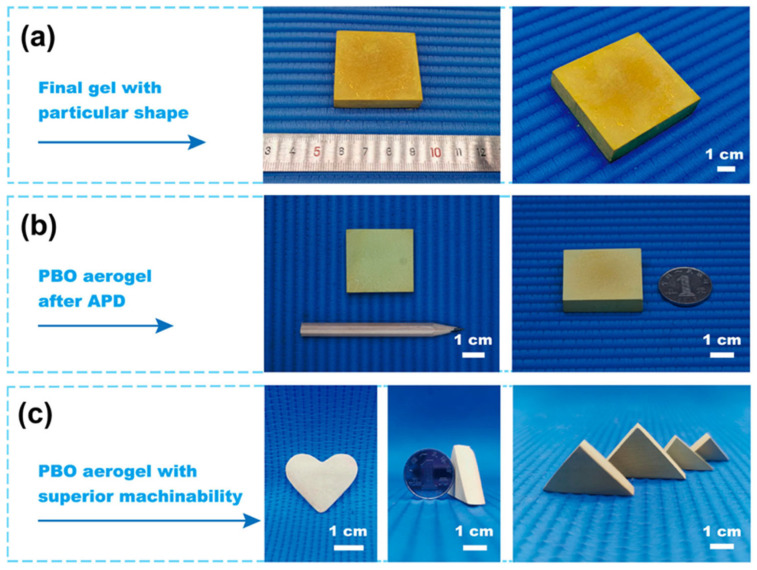
Final PBO gels with particular shapes (**a**) PBO aerogel, (**b**) PBO aerogel after APD and (**c**) PBO aerogel after cutting into desired shapes [1].

PBO-AH exhibited superior thermal insulation compared to conventional plastic insulation materials and displayed remarkable self-extinguishing properties, instantly extinguishing itself when exposed to a 1200 °C flame. Additionally, its thermal insulation was evident from its ability to maintain a cold surface temperature of 36.4 °C after 400 s, even with the opposite surface exposed to a 65.8 °C atmosphere. Furthermore, the aerogels were easily machinable, enabling the formation of custom-shaped samples, underscoring their versatility and practical applications (Figure 8 and Figure 9).

Qin et al. [72] investigated the influence of curing temperature (Tc) on the properties of polybenzoxazine (PBO) aerogels, highlighting that their compressive strength significantly exceeded that of other PBO aerogels of similar density reported in the literature. The study focused on robust F-type PBO aerogels, achieving ultra-high Young’s modulus values of 733.7 MPa at 0.48 g/cm^3^ and 1070 MPa at 0.57 g/cm^3^. These properties were achieved using a straightforward, scalable, HCl-catalyzed sol–gel method at room temperature, followed by ambient pressure drying. The findings revealed that Tc critically impacts the polymerization process and the development of the 3D porous network. At 150 °C, the necks between nanoparticles thickened and strengthened, transforming the microstructure from a “pearl necklace” to a “worm-like” configuration. This transformation enhanced the mechanical properties significantly. However, when Tc exceeded 180 °C, pore volume and specific surface area decreased sharply due to sintering effects, where spherical particles began to agglomerate, as observed in PBO-200 samples. The optimal curing temperature was determined to be 180 °C, where the PBO aerogels exhibited an impressive Young’s modulus of 831 MPa at a density of 0.53 g/cm^3^. At this temperature, the aerogels also achieved a high residual char rate of 62%, making them suitable as precursors for char aerogels. Furthermore, the aerogels displayed excellent thermal insulation properties, with a thermal conductivity of 0.086 W/m·K.

## 3. PBZ Aerogels Derived from Different Benzoxazine Precursors

Xiao et al. [22] synthesized polybenzoxazine (PBO) aerogels using a 4,4′-diamino-diphenylmethane (MDA) benzoxazine monomer through HCl-catalyzed ring-opening polymerization at room temperature, followed by CO_2_ supercritical drying. The resulting aerogels featured a crosslinked three-dimensional network with pore sizes ranging from 10 to 140 nm. This network structure, resembling strings of beads, became denser with increasing aerogel density. The aerogels exhibited thermal conductivities of 0.035–0.057 W/m·K under ambient conditions, which reduced significantly to 0.009 W/m·K at 3 Pa, underscoring their exceptional thermal insulation capabilities. The nanoporous structure effectively encloses gas in tiny spaces, minimizing convective heat transfer when pore sizes are below 1 mm. The aerogels also displayed remarkable mechanical properties, primarily due to their crosslinked structure strengthened by intramolecular hydrogen bonds. The compressive strength of the PBO aerogels, even at lower densities, was substantially higher than that of fiber-reinforced silica aerogels. For instance, at a density of 0.40 g/cm^3^, the PBO aerogels achieved a compressive strength of 14.42 MPa under 10% strain, vastly outperforming silica aerogels reinforced with high-silica glass fibers, which showed a compressive strength of only 1.30 MPa. The study emphasized the critical role of hydrogen bonding in forming the robust three-dimensional network structure, enabling the PBO aerogels to combine high thermal insulation with superior mechanical strength.

**Figure 10:**
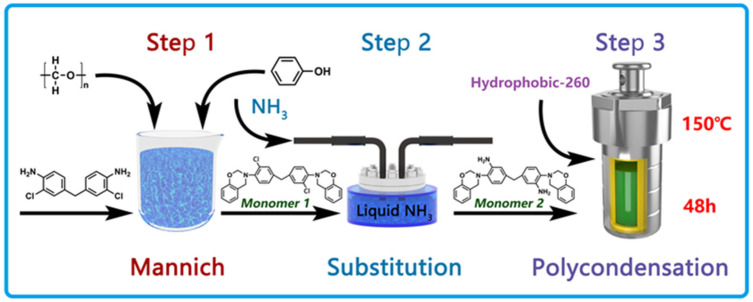
Experimental procedure of polybenzoxazine wet gels [27].

Long et al. [27] synthesized polybenzoxazine (PBO) aerogels using 3,3-dichloro-4,4′-diaminodiphenylmethane, polyoxymethylene, and phenol as raw materials. The process involved the Mannich reaction, liquid ammonia substitution at room temperature under high pressure, and thermally induced ring-opening polymerization (Figure 10).

The introduction of aromatic amines through liquid ammonia substitution provided additional hydrogen donors, enabling the formation of a more extensive and diverse system of hydrogen bonds. These reversible sacrificial hydrogen bonds were found to significantly enhance the mechanical properties of the aerogels. Specifically, the compressive strength of the resulting aerogel (2NPBA) reached 14.36 MPa at a fracture strain of 19.6%, demonstrating notable robustness. This study highlights the critical role of hydrogen bond networks in improving the mechanical performance of PBO aerogels and advancing their application potential in fields requiring materials with both high strength and flexibility.

## 4. PBZ Aerogels Incorporating Inorganic Materials

**Figure 11:**
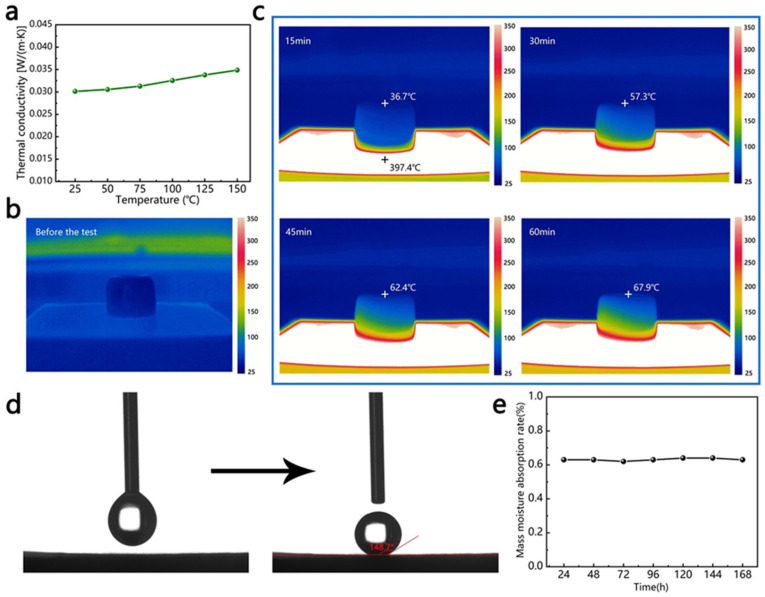
Characterization of the application properties of the 2NPBA. (**a**) Evolution of thermal conductivity with temperature; (**b**,**c**) optical and infrared images of the 2NPBA on a 400 °C heating stage for 60 min; (**d**) instantaneous hydrophobic angle; (**e**) evolution relationship of mass moisture absorption rate with time [27].

Long et al. [27] introduced the innovative concept of reversible sacrificial hydrogen bonding to synthesize a polybenzoxazine (PBO) aerogel with remarkable mechanical strength and a lightweight structure. The aerogel, characterized by a low density of 0.231 g/cm^3^ and a high porosity of 90.7%, demonstrated an exceptional compressive strength of 14.36 MPa and a deformation capacity of 19.6% fracture strain, along with notable fatigue resistance. The aerogel retains 70% of initial maximum stress and 98% of initial height, even after 100 cycles of the compression test at 8% strain. The structural properties of the aerogel, including its low density, high porosity, and mesoporous architecture (average pore size of 24.75 nm) contribute to its excellent thermal insulation performance. Its thermal conductivity values were measured at just 0.03012 W/m·K at 25 °C and 0.03486 W/m·K at 150 °C, making it highly effective for insulation applications. Additionally, the incorporation of hydrophobic groups and doping with superhydrophobic fumed SiO_2_ nanopowders enhanced its hydrophobic characteristics, achieving a contact angle of 148.7° and a minimal saturated mass moisture absorption rate of approximately 0.63% (Figure 11). These combined features—lightweight structure, high mechanical strength, thermal insulation, and superhydrophobicity—underscore the potential of this aerogel for advanced applications requiring multifunctional performance.

**Figure 12:**
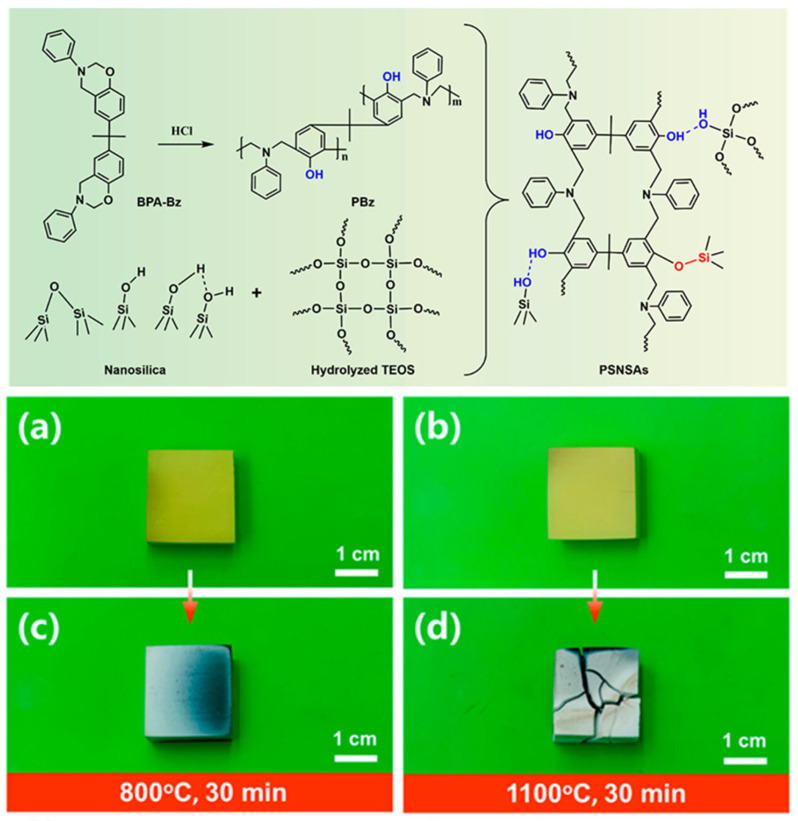
[**Top**] PSNSAs synthesis -mechanism and [**bottom**] PSNSAs-14 samples before heat treatment (**a**,**c**) and after heat treatment (**b**,**d**) in tube furnace [8].

Zhang et al. [8] introduced a novel strategy to enhance the thermal insulation and high-temperature resistance of polybenzoxazine (PBO) aerogels by incorporating a nanoporous silica-phase structure into their network (Figure 12). The nano silica exhibited excellent compatibility with polybenzoxazine, forming a denser nanopore network through chemical bonding. The resulting aerogels displayed remarkable thermal stability, retaining 61.14% of their mass at 800 °C in an oxygen atmosphere. Even after exposure to 800 °C for 30 min, the aerogels maintained their original shape, reflecting exceptional dimensional stability, with a compressive stress of 1.323 MPa under 2% strain.

Post hydrophobic treatment, the water contact angle increased from 0° to 134°, significantly mitigating the degradation of thermal insulation performance caused by moisture absorption. The small pore size and continuous three-dimensional porous structure effectively suppressed gas-phase heat transfer, resulting in reduced heat transfer rates. This robust mesh skeleton endowed the PBO aerogels with excellent thermal conductivity, as demonstrated by a temperature difference of up to 80.9 °C between the hot and cold surfaces in stabilized conditions, showcasing the superior thermal insulation performance of the PSNSAs-14 (PBO/nanosilica aerogels with 14 g of vapor-phase silicon oxide). These findings highlight the potential of this method for advancing the development of high-performance aerogel ablative materials with superior residual capacity and long-term resistance to oxidation, making them suitable for extreme environments.

**Scheme 2:**
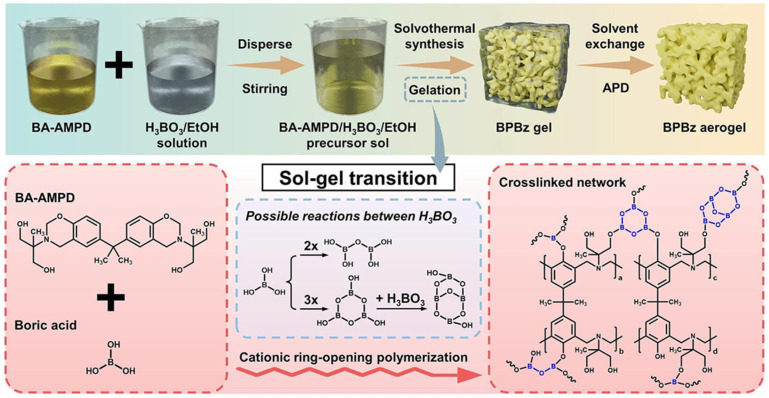
Preparation routes and chemical structure variation of BPBz aerogels [79].

Zhou et al. [79] introduced a boric acid-induced microstructure regulation strategy to fabricate boron-doped polybenzoxazine (BPBz) aerogels, utilizing ethanol as a solvent (Scheme 2). The inclusion of boric acid enhanced the structural integrity of the aerogels by creating intermolecular bridged structures between PBz chains, enabling ambient pressure drying.

**Figure 13:**
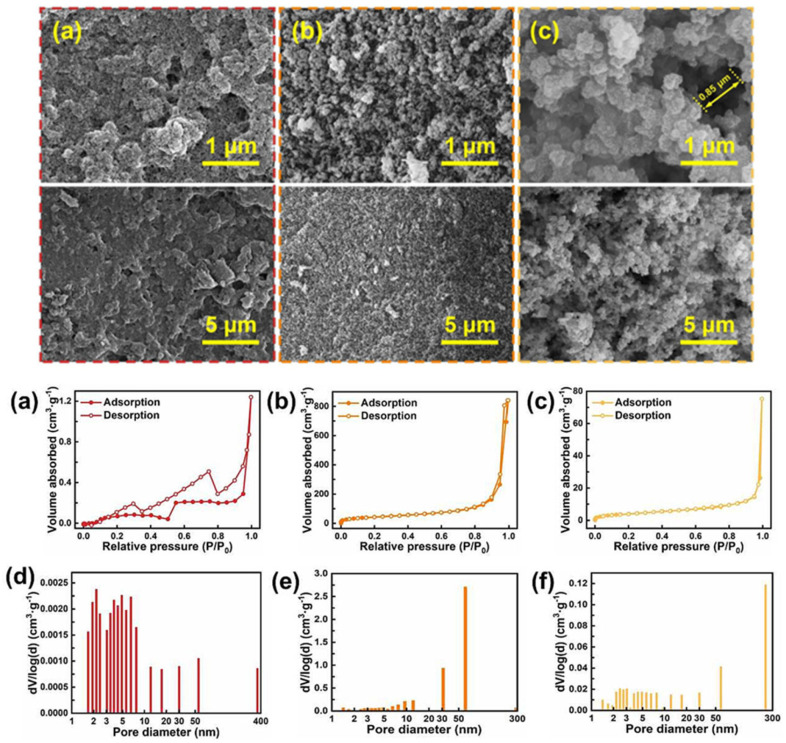
[**Top**] (**a**–**c**) SEM images of BPBz-0, BPBz-1, and BPBz-2 at different magnifications. [**Bottom**] (**a**–**c**) Nitrogen-sorption isotherms and (**d**–**f**) pore size distributions of BPBz aerogels [79].

This approach allowed the aerogels to withstand the capillary forces during solvent drying, preserving their dimensional stability. Both volumetric shrinkage and bulk density decreased significantly with an increasing c/m value, reflecting effective microstructural optimization. The microstructure analysis revealed that an extremely dense state was observed for BPBz-0, whereas, for BPBz-1 and BPBz-2 containing boric acid, a spherical nanoparticle aggregation structure was observed. Moreover, the N2 adsorption isotherms were completely different for the three materials, in which BPBz-2 had large pores of around 300 nm in size (Figure 13). The resulting BPBz aerogels exhibited impressive properties, including high stiffness (specific modulus of up to 255.97 kN·m·kg^−1^), low bulk density (0.203 g/cm^3^), and excellent thermal insulation (thermal conductivity of 0.043 W/m·K). They demonstrated exceptional fire resistance, withstanding exposure to a 1200 °C flame and achieving self-extinguishment in under 1 s. Notably, boron-containing samples (BPBz-1 and BPBz-2) consistently self-extinguished within 1 s in three consecutive flame exposure tests, underscoring the contribution of boric acid to their enhanced flame-retardant properties. Additionally, these aerogels exhibited intrinsic and tunable wettability, which was closely linked to their morphology.

## 5. Hybrids Aerogels and Composites of PBZ

Zhang et al. [4] proposed a method for fabricating polybenzoxazine/silica hybrid aerogels by introducing silica into the polymer matrix through various preparation routes. These hybrid aerogels exhibit remarkable thermal insulation, self-extinguishing behavior, and thermal stability. The polybenzoxazine/silica aerogels (PSAs) demonstrated a mass residual rate of up to 40.19%, primarily due to the robust network structure formed by the silica component, which remains stable under high-temperature oxidizing conditions. PSAs exhibit a higher compressive stress of 3.62, 6.58, and 7.74 MPa, respectively, at strains of 3, 5, and 10%, when compared with PSCAs (0.13, 0.35, and 0.65 MPa). This is due to the fact that PSAs possess enhanced construction without CTS composition, and the addition of CTS in PSCAs weaken their skeleton network. The pore size distribution of polybenzoxazine/silica/chitosan aerogels (PSCAs) predominantly falls within the 5–35 nm range, contributing to their low thermal conductivity of 0.037 W·m^−1^·K^−1^. Compared to PSCAs, PSAs possess smaller pores and more compact skeletal structures, which are critical in adjusting their bulk density and thermal conductivity. Both PSAs and PSCAs display excellent self-extinguishing properties, attributed to the abundant aromatic ring structures in polybenzoxazine and the inclusion of silica as an inorganic phase. The low thermal conductivity of PSCAs is largely due to their entangled and crosslinked nanostructures, which effectively reduce both gaseous and solid heat transfer. Moreover, the addition of chitosan enhances the thermal performance by increasing the pore volume within the 5–35 nm range, further reducing the thermal conductivity compared to PSAs.

**Figure 14:**
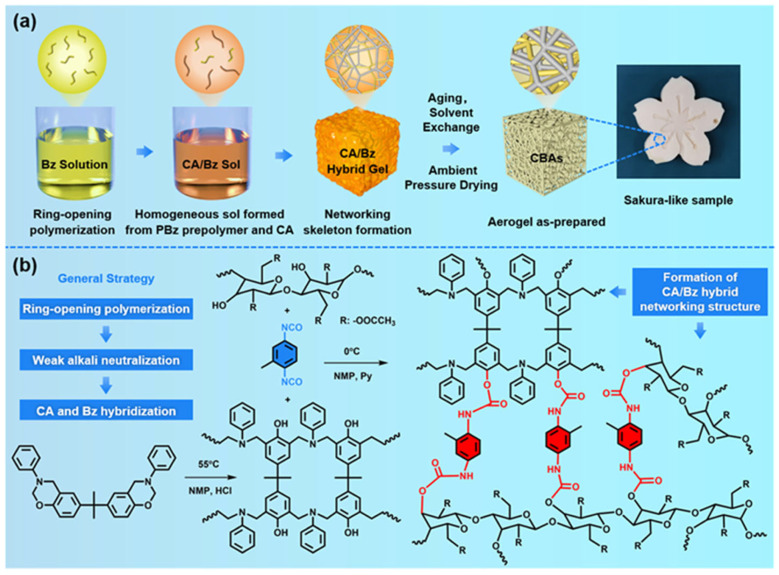
Chemical formation mechanisms of CBA series. (**a**) Fabrication process and (**b**) the reaction mechanism for the ring-opening polymerization reaction of Bz, including the CA and PBz hybrid course [80].

In a subsequent study by the same author [80], a novel and cost-effective method for fabricating cellulose acetate/benzoxazine hybrid aerogels (CBAs) was developed using ambient pressure drying (Figure 14).

These CBAs exhibit low drying shrinkage, excellent mechanical performance even under cryogenic conditions (−196 °C), superior thermal insulation, flame retardancy, and high thermal stability. The weighted drying shrinkage rate of CBAs-T2 (‘T’ represents different contents of TDI—toluene diisocyante) was reduced to 6.8% (averaged across radial and axial directions), mainly due to the strengthened framework created by the incorporation of polybenzoxazine network chains. CBAs-T2 demonstrated remarkable mechanical strength at room temperature, which can be attributed to the hybrid structure formed between the polybenzoxazine and cellulose networks. Notably, these aerogels retained their mechanical properties even after exposure to liquid nitrogen, highlighting their robustness in extreme conditions. The optimized CBAs-T2 achieved a low thermal conductivity of 0.033 W·m^−1^·K^−1^, attributed to their uniform crosslinked network and fine pore structure. In terms of flame retardancy, CBAs-T2 exhibited self-extinguishing behavior upon ignition, which is largely due to the aromatic rings present in the polybenzoxazine backbone. Furthermore, the aerogels’ excellent thermal stability was linked to the ability of polybenzoxazine to resist cellulose acetate decomposition and suppress heat release during combustion, contributing to their enhanced fire resistance.

The integration of porous hosts with organic phase change materials (PCMs) enables efficient thermal energy storage and multifunctional applications. However, this combination is typically flammable due to the low carbonization of PCMs and the porous structure, which facilitates oxygen diffusion and combustion. Host–guest composites, combining porous scaffolds with organic PCMs, offer high energy density and customizable functions, making them promising for advanced thermal storage systems. Nonetheless, traditional flame-retardant methods often struggle to balance fire safety with the preservation of latent heat in these materials. To address this challenge, Liu et al. [81] developed a high-temperature-triggered crosslinking strategy by utilizing a polybenzoxazine-based aerogel (PB-1) as the host and benzoxazine-based PCMs (C-dad, benzoxazine monomer with long fatty chain) as the guest. At elevated temperatures, the C-dad undergoes ring-opening polymerization (ROP) initiated by the phenolic groups of PB-1, forming a crosslinked polybenzoxazine copolymer monolith. This reaction enhances the char yield and inherently reduces flammability without relying on conventional flame-retardant additives. The resulting composite (PB-1/C-dad) achieves an optimal balance between latent heat (145.3 J·g^−1^), char yield (13.1% residue at 600 °C), and flame retardancy (peak heat release rate of 231 W·g^−1^). These properties surpass those of many flame-retardant modified polymer/organic PCM systems reported in the literature, demonstrating the effectiveness of this novel crosslinking approach in improving fire safety while maintaining high thermal performance.

Liu et al. [12] developed high mass residual polybenzoxazine/silica (PBOS) aerogels through the in situ copolymerization of benzoxazine prepolymers with N,N-dimethylformamide (DMF)-soluble silica (SiO_2_) precursors. The copolymerization process resulted in dual-network nanoporous structures, formed due to the synchronized gelation and crosslinking rates of polybenzoxazine (PBO) and SiO_2_. The addition of ethanol and an elevated gelation temperature played key roles in achieving these structures. The PBOS aerogels demonstrated significantly enhanced mass retention compared to traditional silicon-modified PBO aerogels, with residual masses of 75.96% in argon (an increase of ~26%) and 52.23% in air (an increase of ~30%). Notably, the PBOS-4 aerogel exhibited exceptional thermal insulation performance, maintaining a back temperature of only 59.9 °C when exposed to a 1200 °C butane flame for 600 s. Thanks to the high mass residuals and superior thermal stability, the quartz fiber-reinforced PBOS-4 composite (QF/PBOS-4) showed remarkable ablation resistance. When subjected to a 1500 °C oxyacetylene flame for 600 s, it achieved linear and mass ablation rates of just 0.8 μm·s^−1^ and 0.67 mg·s^−1^, respectively, demonstrating its excellent durability under extreme thermal conditions. Comparative data showing various properties, including density, porosity, thermal conductivity, mechanical strength, and flame retardancy of different PBZ aerogels are given in Table 2.

## 6. Conclusions and Future Perspectives

Polybenzoxazine aerogels are an emerging class of thermal insulation materials known for their superior thermal stability, low thermal conductivity, flame retardancy, and customizable properties (Table 2). They are synthesized through the polymerization of benzoxazine monomers, resulting in nanoporous structures that efficiently insulate heat while maintaining mechanical integrity under harsh conditions. Recent research shows that performance enhancements in polybenzoxazine aerogels are achieved by incorporating silica phases, hybridizing with other polymers, and introducing flame-retardant mechanisms without conventional additives. These advancements enable the aerogels to withstand high-temperature flames while maintaining structural integrity, making them ideal for aerospace, automotive, and building industries. A key innovation is the development of dual-network structures like polybenzoxazine/silica hybrids, which offer high char yields and superior ablation resistance. Additionally, the incorporation of crosslinking mechanisms and reinforcing fibers has further enhanced their mechanical strength and fire resistance, positioning polybenzoxazine aerogels as competitive alternatives to traditional materials like silica aerogels and polymer-based composites.

PBZ aerogels derived from bisphenol-A raise environmental concerns, as bisphenol-A is a potential carcinogen. To address this, the molecular design flexibility of benzoxazine monomers allows for alternative synthesis approaches, such as modifying the phenol component or producing bio-based benzoxazines. The production of PBZ aerogels involves three key steps: the use of solvents for gelation, achieving uniform gelation, and employing supercritical CO_2_ for drying. Green solvents like ethanol or water are preferable, as they help to mitigate environmental contamination. Uniform gelation is crucial to ensure homogeneity in porosity, which ultimately determines the aerogel’s properties. However, the reliance on supercritical CO_2_ for drying is energy-intensive and requires specialized equipment, posing cost and scalability challenges. Developing ambient pressure drying methods could significantly reduce production costs and improve scalability. For PBZ aerogels combined with inorganic materials and composites form PBZ aerogels, functionalization to enhance properties such as thermal and mechanical stability and flame retardancy introduces additional complexity. Future research should prioritize green synthesis routes, ambient drying techniques, and low-energy alternatives to enhance cost-effectiveness, minimize environmental impact, and facilitate large-scale industrial production. Key goals include reducing density while maintaining thermal performance using techniques like freeze-drying or 3D printing. To ensure commercial viability, scalable and cost-effective production methods—such as solvent-free polymerization or ambient pressure drying—must be developed. Innovative designs and efficient manufacturing will enable the broader adoption of these aerogels in advanced thermal management applications.

## Figures and Tables

**Table 1:** Properties of aerogels, cryogels, and xerogels.

Property	Aerogels	Cryogels	Xerogels
Porosity	Very high (99%)	High (90–95%)	Moderate (70–80%)
Surface area	Highest (500–1200 m^2^/g)	Moderate (10–300 m^2^/g)	High (100–800 m^2^/g)
Thermal insulation	Excellent (0.013–0.02 W/m·K)	Poor (0.04–0.06 W/m·K)	Moderate (0.03–0.05 W/m·K)
Mechanical properties	Brittle	Flexible but weaker than aerogels	Weak and prone to cracking
Production cost	High (supercritical drying)	Moderate (freeze-drying)	Low (ambient drying)
Scalability	Limited	Moderate	High

**Table 2:** Comparison data showing the property of different PBZ aerogels.

Material	Density (g/cm^3^)	Porosity/ Pore Size/Volume	Thermal Conductivity (W/m·K)	Mechanical Strength	Flame Retardancy
PBO aerogels [38]	0.084	60–70 nm	0.0335	-	-
F-20 [50]	0.2815	10–50 µm	0.0604	Compressive strength: 6.97 MPa	-
PBO-7 [65]	0.24	80.92%	0.097	Young’s modulus: 28.90 MPa	Self extinguishable and stabilized after 1 min
PBO-AH [1]	-	40–60 nm	0.040	Compressive stress: 2.98 Mpa at 3% strain	Self-extinguishable immediately when exposed to a 1200 °C flame
PBO-180 [72]	0.531	22.64 nm	0.086	Compressive stress: 23.85 Mpa at ultimate strain	Self extinguished instantly when exposure to the alcohol lamp (≈660 °C).
PBO-5.0 [22]	0.108	55 nm	0.011	Compressive strength: 14.42 MPa	-
2NPBA [27]	0.231	90.7%	0.03012	Compressive strength: 14.36 MPa	-
PSNSAs-14 [8]	0.49	16.91 nm	0.0491	C = 1.323 MPa	Self-extinguished within 1 s when exposed to a 1200 °C flame
BPBz-2 [79]	0.203	300 nm	0.043	Specific modulus of up to 255.97 kN·m·kg^−1^	Self-extinguished within 1 s when exposed to a 1200 °C flame
PSA [4]	0.40	5–35 nm	0.064	Compressive stress: 3.62 Mpa at 3% strain	Self-extinguishable immediately when exposed to a 1200 °C flame.
PSCA [4]	0.26	5–35 nm	0.037	Compressive stress: 0.13 Mpa at 3% strain	Self-extinguishable immediately when exposed to a 1200 °C flame.
CBA-T2 [80]	0.11	30.60 nm	0.033	Compressive stress: 0.73 Mpa at 3% strain	Self-extinguishable within few seconds when exposed to a 1200 °C flame.
PB-1/C-dad [81]	0.14	Pore volume: 0.5 cm^3^/g	Total heat realease: 42.2 kJ/g	-	Peak heat release rate of 231 W·g^−1^
PBOS-4 [12]	0.405	10 nm	0.0699	Compressive strength: 110.9 MPa	Exposed to a 1200 °C butane flame for 600 s
QF/PBOS-4 [12]	-	-	0.057	Compressive strength: 16.9 MPa	Linear and mass ablation rates of just 0.8 μm·s^−1^ and 0.67 mg·s^−1^

## Data Availability

Not applicable.
